# The Multimodal Assessment of Adult Attachment Security: Developing the Biometric Attachment Test

**DOI:** 10.2196/jmir.6898

**Published:** 2017-04-06

**Authors:** Federico Parra, Raphaële Miljkovitch, Gwenaelle Persiaux, Michelle Morales, Stefan Scherer

**Affiliations:** ^1^ Institute for Creative Technologies University of Southern California Los Angeles, CA United States; ^2^ Paragraphe Laboratory Paris VIII University Saint-Denis France; ^3^ University Hospital Center Sainte-Étienne Sainte-Étienne France; ^4^ The Graduate Center City University of New York New York, NY United States

**Keywords:** psychometrics, linguistics, heart rate, facial expression, psychophysiology, psychopathology, COVAREP, attachment

## Abstract

**Background:**

Attachment theory has been proven essential for mental health, including psychopathology, development, and interpersonal relationships. Validated psychometric instruments to measure attachment abound but suffer from shortcomings common to traditional psychometrics. Recent developments in multimodal fusion and machine learning pave the way for new automated and objective psychometric instruments for adult attachment that combine psychophysiological, linguistic, and behavioral analyses in the assessment of the construct.

**Objective:**

The aim of this study was to present a new exposure-based, automatic, and objective adult-attachment assessment, the Biometric Attachment Test (BAT), which exposes participants to a short standardized set of visual and music stimuli, whereas their immediate reactions and verbal responses, captured by several computer sense modalities, are automatically analyzed for scoring and classification. We also aimed to empirically validate two of its assumptions: its capacity to measure attachment security and the viability of using themes as placeholders for rotating stimuli.

**Methods:**

A total of 59 French participants from the general population were assessed using the Adult Attachment Questionnaire (AAQ), the Adult Attachment Projective Picture System (AAP), and the Attachment Multiple Model Interview (AMMI) as ground truth for attachment security. They were then exposed to three different BAT stimuli sets, whereas their faces, voices, heart rate (HR), and electrodermal activity (EDA) were recorded. Psychophysiological features, such as skin-conductance response (SCR) and Bayevsky stress index; behavioral features, such as gaze and facial expressions; as well as linguistic and paralinguistic features, were automatically extracted. An exploratory analysis was conducted using correlation matrices to uncover the features that are most associated with attachment security. A confirmatory analysis was conducted by creating a single composite effects index and by testing it for correlations with attachment security. The stability of the theory-consistent features across three different stimuli sets was explored using repeated measures analysis of variances (ANOVAs).

**Results:**

In total, 46 theory-consistent correlations were found during the exploration (out of 65 total significant correlations). For example, attachment security as measured by the AAP was correlated with positive facial expressions (*r*=.36, *P*=.01). AMMI’s security with the father was inversely correlated with the low frequency (LF) of HRV (*r*=−.87, *P*=.03). Attachment security to partners as measured by the AAQ was inversely correlated with anger facial expression (*r*=−.43, *P*=.001). The confirmatory analysis showed that the composite effects index was significantly correlated to security in the AAP (*r*=.26, *P*=.05) and the AAQ (*r*=.30, *P*=.04) but not in the AMMI. Repeated measures ANOVAs conducted individually on each of the theory-consistent features revealed that only 7 of the 46 (15%) features had significantly different values among responses to three different stimuli sets.

**Conclusions:**

We were able to validate two of the instrument’s core assumptions: its capacity to measure attachment security and the viability of using themes as placeholders for rotating stimuli. Future validation of other of its dimensions, as well as the ongoing development of its scoring and classification algorithms is discussed.

## Introduction

### The Relevance of Adult Attachment in Mental Health

Attachment theory originated with the work of a British psychiatrist, John Bowlby [1]. Inspired by ethological observations and evolution theory, he theorized that the chance for survival of human genes had increased by the natural selection of behaviors that augmented proximity and bonding between infants and their caregivers, leading to a greater probability of protection for the children [1,2]. Attachment theory posits an innate psychobiological behavioral system, the *attachment system*, which activates specially in times of perceived threat, inciting the child to seek the proximity and care of their caregivers, the *attachment figures*. The system deactivates once a felt sense of security and safety is reestablished [1,3]. Despite the universality of attachment proximity-seeking behaviors in children [4,5], the security and care sought are only found when the attachment figures are capable of responding promptly and adequately [6]. The quality and outcome of these repeated early attachment interactions leave an enduring mark in the developing person [7-10]. The nature of this mark is threefold: it is cognitive, since dynamic representational models of the attachment figures and the relationship with them develop [11,12], contributing in adulthood to appraisals of the self as worthy of care and of others as capable of providing care [13]; behavioral, because our innate attachment behaviors accommodate to the environment [6], for example, in case of continuous unavailability of caregivers children might stop proximity-seeking behaviors entirely and act as if they were totally independent, a pattern that is then carried into adulthood [14]; and psychobiological, because negative early attachment experiences can lead to differences in the response of the bilateral amygdala and left ventral striatum during stressful situations, and to an overall higher sympathetic activation baseline [15-17].

The different adult attachment patterns have been extensively described in the literature using both dimensional and categorical models [2,18]. In the dimensional approach, the single most important dimension is *attachment security* [12].

Attachment theory sparked some of the largest and more rigorous longitudinal studies in psychology to date [8,19], proving itself essential in three overlapping research domains of mental health: the study of psychopathology, the study of psychological development, and the study of the psychology of adult interpersonal relationships.

In terms of clinical research, longitudinal studies have shown that negative early attachment interactions in childhood predict childhood attachment security [9], which in turn partially predict adult psychopathology [7,20], whereas cross-sectional studies have consistently linked adult *attachment insecurity* to several psychopathologies [21,22], such as depression [23], post-traumatic stress disorder (PTSD) [24], or borderline personality disorder [25]. Positive attachment experiences in adulthood, whether naturally occurring or the outcome of therapeutic interventions, can help increase attachment security, which in turn improves mental health [26-28].

In terms of developmental psychology, studies show that developmental competencies that are essential to sustain mental health and to cope with mental health disorders, such as emotional regulation, social skills, or cognitive ability, are associated and interdependent with attachment across the lifespan [7,29,30].

Finally, adult attachment is key in the psychology of interpersonal relationships, including long-term romantic relationships [31,32], which tend to function as a buffer in coping with psychopathology and stress [33,34]. Attachment insecurity has been associated with having more interpersonal problems in general [35], and these problems explain insecure persons’ self-reported loneliness, social isolation, low relationship satisfaction, more frequent relationship breakups, greater physiological stress reaction to interpersonal conflict, and more frequent conflicts and violence [21,36-38]. Secure attachment, in relationship with social support, has been acknowledged as a protective factor for psychological stress [34], with perceived social support mediating the relationship between attachment security and depressive symptoms [33].

### Current Limitations in the Assessment of Adult Attachment

Since 1985 (Findings by George C, Kaplan N, and Main M, unpublished data, 1985), various validated instruments for the assessment of adult attachment developed concomitantly within the fields of social psychology and developmental psychology (for a review, see [39]). Social psychology has spurred the development of several questionnaires, such as the Adult Attachment Questionnaire (AAQ [40]) or the Adult Attachment Scale (AAS [41]). Developmental psychology, on the other hand, has relied on a variety of broadly defined semistructured interview methods, beginning with the Adult Attachment Interview (AAI; Findings by George C, Kaplan N, and Main M, unpublished data, 1985) which is considered the “gold standard” [39]. Both approaches suffer from several limitations that affect both research and clinical assessments, and that are reflective of the current state of psychometrics.

Questionnaire-based assessments are self-report measures. As such, they are prone to self-report biases that have been well described in the literature [42]. In terms of construct validity, there has been no longitudinal association demonstrated between attachment in childhood as measured for example with the Strange Situation Procedure (SSP [43]) and adult attachment as measured with questionnaires [19]. Furthermore, almost no concurrent validity has been found between questionnaires and interview-based assessments of adult attachment, adding to the construct validity controversy [39]. On the other hand, questionnaires of adult attachment are easy, economic, and fast to both administrate and score. Administration can be done remotely, and automatic scoring is possible. These positive practical psychometric characteristics may explain the surge of studies that have chosen questionnaires of adult attachment as their measure [39].

Interview-based assessments of adult attachment rely on some form of semistructured interview, which is later transcribed and scored by a trained judge, that has undergone substantial training in a specific standardized scoring tradition. In a way, this form of assessment is closer to child assessments which also rely on third-party experts for scoring and classification. However, in childhood-attachment assessments such as the SSP, the scoring experts observe behavior in general, whereas in interview methods only transcribed language is analyzed during scoring, thus limiting the scope of dimensions evaluated in this process. The Attachment Multiple Model Interview (AMMI [12]) circumvents this limitation in part, by including in the interview specific probes to gather self-reported information about behaviors.

In terms of construct validity, the AAI has consistently shown a link between parents and their children’s attachment patterns, which is considered strong evidence of its validity [2]. Moreover, a substantial longitudinal link has been found using the AMMI [12], further supporting the consensus that interview methods based on expert judgment can produce results with higher construct validity than self-report measures.

But despite this consensus, interview methods are not without their own limitations. In contrast to their questionnaire-based counterparts, interview methods are difficult, costly, and lengthy to both administer and score. They add additional layers to the process, that is, the manual transcription and coding of the interview. There is a training load required for both administrating and scoring. This process is costly.

Interview methods are impacted by an additional problem: the subjectivity inherent to an expert judge [44]. This limitation might decrease the replicability of attachment studies, adding to psychology’s current “replicability crisis” [45].

Finally and contrary to questionnaires, interview methods cannot be administered remotely, limiting their application, for instance, in Internet-based research.

### Advances in Multimodal Analysis and Automatic Detection of Psychological Markers

Finding psychophysiological and behavioral markers of psychological conditions is gaining traction within mainstream psychiatry [46,47], as part of a quest to provide more objective and precise clinical assessments to patients. The American National Institute of Mental Health released a statement in 2013 [48] in which it made explicit its desire of moving toward more objective and precise diagnostic methodologies. Several attempts to tackle this problem have arisen from the Computer Sciences. In a recent review, Cummins et al [46] reviewed the state-of-the-art in the automatic detection of depression and suicidality through the analysis of speech and its paralinguistic acoustic features. Scherer et al [49] described, in 2013, a set of automatically extracted audiovisual nonverbal behavioral features helpful in the identification of depression, anxiety, and PTSD [49]. The link between the objective measure of singular biometric or behavioral markers, and the sought ability to offer more precise diagnoses, relies on the use of machine learning algorithms that can fusion multiple modalities of data at once [50]. This allows for the uncovering of complex multimodal data patterns that can serve in the automatic assessment of specific mental conditions. In recent studies, such multimodal systems have approached human performance in the detection of indicators of PTSD [51]. Since several studies on the specific psychophysiological [2] and linguistic [44] traits of adult attachment already exist and show promise, we decided it was time to use this new technology in the assessment of adult attachment.

### The Biometric Attachment Test

The Biometric Attachment Test (BAT) was created with the objective of automatically and objectively measuring attachment in adults. At its core, the BAT is an exposure-based test, which means that the participant being tested is exposed to a short (9 min) standardized set of visual and music stimuli, whereas their immediate reactions and verbal responses, captured by several computer sense modalities, are automatically analyzed for scoring and classification.

There are two aspects of the development of the BAT that require separate attention: the instrument itself, meaning its assumptions, stimuli selection, and administration protocols, which will be articulated in this work; and the test’s automatic classification and scoring algorithms, a work-in-progress that we will briefly touch upon in “Discussion” section.

### Construction of the Biometric Attachment Test (BAT)

The BAT was strongly influenced by three previous instruments: Bowlby’s first Separation Anxiety Test (SAT [52]), the previously mentioned SSP [43], and the AAP [13].

The SAT (1976 version [52]) is a projective attachment test for children aged 4-7 years consisting of a set of 6 pictures depicting situations in which a child, separated from their family, must cope on their own without help from their parents. The tested child is asked to interpret the protagonist’s feelings and predict their behavior, and their transcribed responses are later scored and classified.

The SSP is a structured observation protocol for assessing attachment in children aged between 12 and 24 months. During 20 min, the child undergoes a series of separations and reunions from their caregiver, while they are also exposed to the arrival and presence of a stranger. The child’s behavior is videotaped and then analyzed for attachment scoring and classification.

The AAP is an adult attachment test based on a set of black and white drawings, some of which are ambiguous, depicting more diverse situations that activate the attachment system: separation, loss, solitude, and physical threat [13]. Participants are asked to tell a short story about the pictures, which are transcribed and analyzed, and an attachment classification and continuous scores are obtained [13].

Like the SAT, our BAT uses photos, of real people, in explicit situations. Like the AAP, our stimuli depict a variety of attachment-sensitive situations. Like the SSP, the BAT is meant to produce an *alternating activation and deactivation* of the attachment system, with stimuli representing themes such as loss, death, or separation alternating with stimuli representing themes such as intimate connection, soothing, or protection.

Unlike other exposure-based and projective tests, the BAT uses music stimuli in addition to visual stimuli, both on its own and concomitantly with visual stimuli. Music was included because of its ability to trigger strong emotional feelings and experiences [53].

Like the SSP, scoring and classification in the BAT take into consideration observed behaviors. In fact, unlike other tests in which verbatim transcripts of verbal responses are analyzed, the BAT captures the participants’ reactions and responses in a variety of modalities: physiological (heart rate [HR] and electrodermal response [EDA]) from which psychophysiological features can be derived (eg, Bayevsky stress index [54]), behavioral (facial expressions, gaze, face distance from stimuli, paralinguistic speech characteristics), and verbal.

### The Concept of Themes in the Biometric Attachment Test (BAT)

Exposure-based and projective psychometric tests typically rely on a fixed set of stimuli selected by the authors [13,55]. We pose the following critiques to this approach: first, stimuli can eventually leak into the public domain, such as in the case of the Rorschach [56], and this might undermine a test’s effectiveness due to priming effects. Second, longitudinal studies such as clinical trials require participants to be tested several times using the same instruments, and if stimuli are always the same this might also lead to priming effects. Finally, we believe ideally stimuli should be selected based on input from the general population toward which it is destined.

Our BAT innovates introducing the concept of *themes*: placeholders for actual stimuli. A theme is a narrative that describes a specific situation to be evoked by a stimulus, with a specific objective. For example, in terms of adult attachment, a theme could be “the loss of a close one,” its objective being to activate the attachment system (ie, to cause attachment-related distress).

Themes thus can solve the aforementioned problems with fixed-stimuli test designs: since themes are placeholders for stimuli as opposed to fixed stimuli, there is no risk if a stimuli set becomes widely known. All the contrary: stimuli in the BAT can—and should—be replaced from time-to-time and from context-to-context. In the case of clinical trials, stimuli sets in the BAT could rotate between assessments. Finally, the process for stimuli selection in the BAT is standardized and crowdsourced, as we will see briefly.

**Figure 1:**
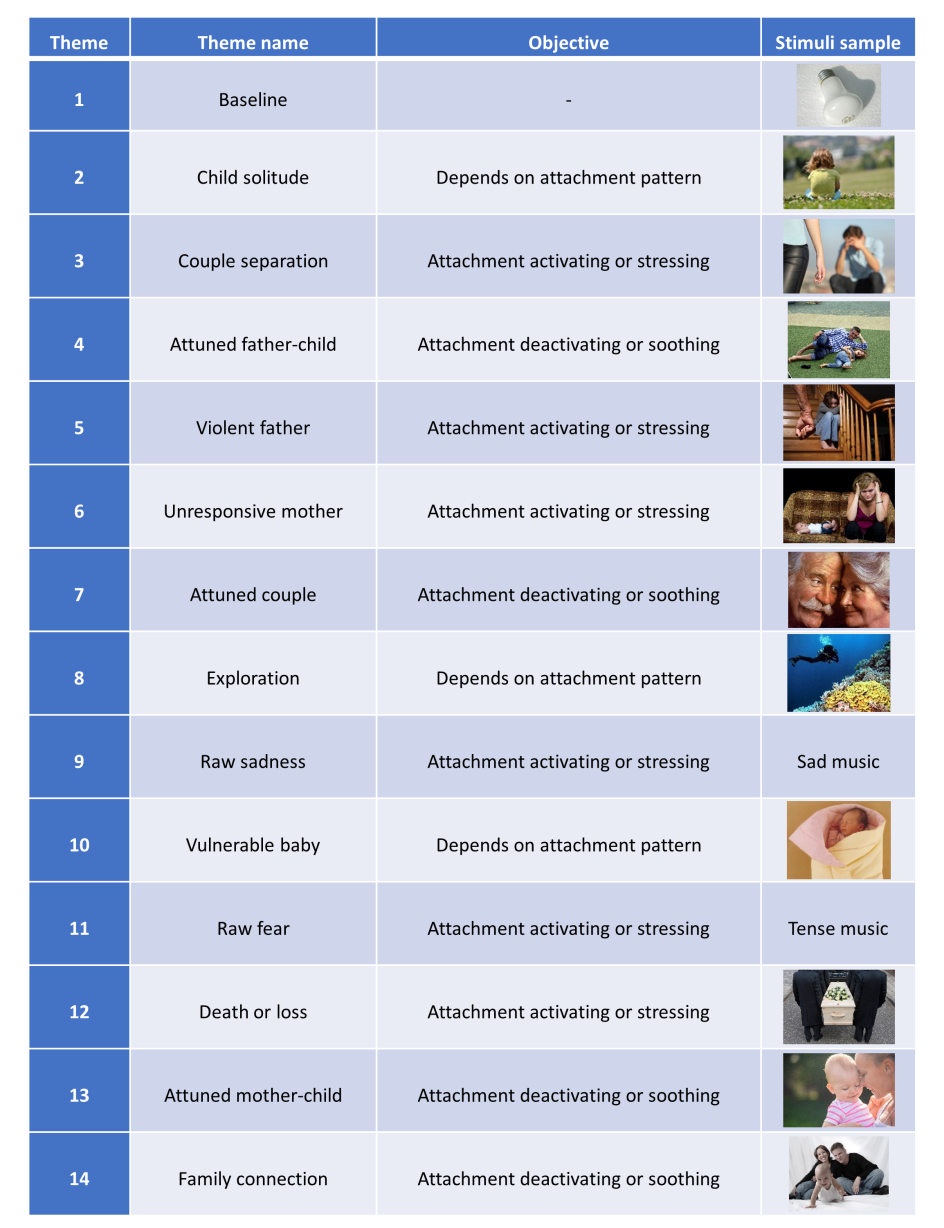
Biometric Attachment Test (BAT) themes, goals, and stimuli set sample.

About the themes’ objectives, each is meant to evoke a reaction in the participant depending on the participant’s attachment patterns. The themes were inspired by the SAT, the AAP, the SSP, and attachment theory core principles. In total, 14 themes resulted from this work (see Figure 1).

Theme 1 (“baseline”) was designed to measure the participants’ reactions to being in the test situation, where they are still not being confronted with any attachment-specific stimulus. This provides proper baselines for all biometric and behavioral measures.

Themes 2, 8, and 10 were designed to elicit specific reactions depending on the underlying attachment pattern of the test participant, to help in classification.

All other BAT themes have per objective to either activate (ie, stress) or deactivate (ie, calm) the attachment system. We would like to clarify that throughout this paper we use the terms “attachment activation” and “attachment deactivation” in their literal sense, that is, the way in which the attachment system is activated when under specific relational stress and how it becomes deactivated when that relational stress is sufficiently addressed. This is not to be confused with “avoidant deactivation,” a “Minimizing strategy (...) conceived by Main (1990) as a shift of attention away from conditions normally eliciting attachment behavior, leading to the apparent absence of attachment behaviors in such circumstances” [12].

### Stimuli Selection in the Biometric Attachment Test (BAT): A Standardized Process

A set of objective and subjective criteria were developed for each of the BAT’s themes. The objective criteria were directly derived from the themes’ narratives: for example, for a stimulus to be appropriate to represent the “attuned mother-child” theme, there should be a mother and a child in the picture. Subjective criteria are notions that require more complex judgments: for example, for a stimulus to be appropriate to depict the “attuned mother-child” theme, the child and the mother must seem attuned to each other and, thanks to said attunement, they should both seem relatively relaxed. To decide whether a mother or a child seem relaxed or not just by looking at them in a picture is a subjective process that should not be arbitrarily decided by researchers.

We used the straightforward objective criteria to preselect stimuli: three large picture databases conceived for the study of emotion were used: the Nencki Affective Picture System (NAPS [57]), the International Affective Picture System (IAPS [58]), and the Geneva Affective Picture Database (GAPED [59]). In some cases, none of these databases had enough pictures for some of the themes, so we turned to a stock picture service, iStockPhoto. We ended up with 126 preselected pictures.

### The Web-Based Survey

We then created an anonymous web-based survey using SurveyGizmo services. The survey randomly introduced each of the preselected pictures, accompanied by sliders that participants could adjust to the right or to the left, signaling a judgment about a specific criterion. In the case of the “attuned mother-child” theme, for example, one of the available sliders allowed the participant to judge the perceived level of stress of the child in the picture. We always opposed two traits (eg, stressed vs relaxed), randomizing their order and starting with the slider in the center among them.

Our survey was made available in Spanish, English, and French and was distributed through social media and email campaigns in the United States, France, and Argentina. A total of 520 participants (female=72.3%, 376/520, male=27.7%, 144/520), of a variety of ages (mean 37.53, SD 10.87) responded. The survey was kept online for a period of 10 days between March 3, 2016 and March 13, 2016. Results where then cleaned-up using standard survey results cleaning best practices [60].

We created composite scores formulas for each of the 14 BAT themes, allowing to combine the subjective criteria measured in the survey. For the “attuned mother-child” theme, for example, the composite score formula was composed by the perceived level of genuineness of the picture, plus the perceived attunement between mother and child in the picture, minus the perceived levels of stress in the child and in the mother in the picture, individually.

A minimum required composite score was set for each theme to prevent stimulus that are not evocative enough from being used in the future.

The list of themes, objective and subjective criteria, as well as the survey design, are available for other researchers to generate new stimuli sets for the BAT in the future (contact corresponding author).

Music selection was easier and did not require a survey process. Music themes were conceived to convey basic raw emotions (eg, theme 9, “raw sadness”). A total of 25 short music clips were selected from a music set conceived to elicit emotion and that already provides scores on discrete perceived emotions [61]. We simply chose the music clips with higher scores in the required emotion per theme.

### Biometric Attachment Test (BAT) Administration Procedure

The BAT automatic administration procedure was constructed using OpenSesame software, version 3.1.2 [62]. The full test duration is of 9 min.

Before beginning, the participant is instructed to observe the visual or listen to the music stimuli as long as it is visible or audible, and then to describe aloud what they felt about it.

During the test, each theme stimulus is automatically presented for 15 s, followed by a black screen displaying the phrase “What did you feel?” which shows for 25 s, whereas a countdown slider displays the available time to respond. It then shows the phrase “Thank you. Here is the following stimulus...,” for 5 s, followed by the following stimulus, and so forth.

The test is administered with the person being alone with the computer in a room; aloneness can facilitate the activation of the attachment system [1] and renders the test situation closer to Ainsworth’s SSP [6]. It also removes possible interference from researchers.

### Hypotheses of This Study

This study was designed to empirically evaluate two core assumptions of the BAT:

H1: Those adults with higher attachment security will more successfully use the BAT’s attachment-deactivating themes to reassure and soothe themselves, and this will be in turn reflected in specific psychophysiological, behavioral and linguistic markers. Theme 4 (“attuned father-child”), 7 (“attuned couple”), and 13 (“attuned mother-child”) are evocative of the availability of attachment figures and will be used to test this hypothesis.

H2: Different stimuli sets, selected through our standardized process, are interchangeable and cause very similar responses or reactions in participants. Specifically, the features most associated with attachment security will remain consistent across three different stimuli sets.

## Methods

### Sample

The sample consisted of 59 French francophone participants (45 females, 14 males) that were interviewed between March and May, 2016. The sample was formed from multiple sources in different regions of France: 9 psychiatric patients recruited at University Hospital Center Sainte-Étienne and 7 recruited at the Ville Evrard Center of Psychotherapy in Saint Denis; 29 volunteers enrolled in Mornant, Paris, and Rouen; and 14 college students enrolled at Paris 8 University in Saint Denis. It was intended for the sample to be as diverse as possible in terms of age (mean 35.7, SD 12.2), occupational status (10% unemployed, 6/59, 32% employed, 19/59, 33% students, 20/59, 23% other, 14/59), as well as relationship status (37% in a relationship, 22/59, 23% married, 14/59, 11% separated or divorced, 7/59, 25% single, 14/59, 3% unknown, 2/59) and psychopathology (27%, 16/59 were patients). Since questions about ethnicity or race are not allowed in French research, we don’t have information to report about the ethnic diversity of our sample. All participants signed informed consent forms in accordance to best practices in French Universities.

### Measures

#### Adult Attachment Questionnaire

Fifty of our participants completed the AAQ before the interview, as a web-based questionnaire. The AAQ is a 17-item measure that asks individuals to indicate how they relate to romantic partners in general. It yields a continuous measure of attachment security with regards to romantic partners [40].

#### Adult Attachment Projective Picture System

All our participants completed the AAP test, which was introduced earlier. Transcripts of the AAP were scored by a trained member of our team blind to all information about the participants. Interjudge reliability was obtained for 5 cases that were double-coded by one of the AAP’s creators, with 80% of interrater agreement for both classifications and scores. The AAP outputs a continuous attachment security score, called “agency of self,” which per George [13] has both an inward and outward aspects. For this study, we’ll focus on the latter, which evaluates the degree to which an individual seeks for, and trust, attachment figures to provide for them a haven of safety in times of stress [13].

#### Attachment Multiple Model Interview

The AMMI is a validated semistructured interview that investigates participants’ reactions in attachment-related situations. By analyzing and scoring transcriptions of the interviews, AMMI provides scores for three different attachment relationships: attachment to mother, father, and partner [12]. Since each relationship requires a specific amount of interview time, not all participants were able to complete all the interviews: attachment to the mother was evaluated for 27 participants, attachment to the father for 23, and attachment to the partner for 17. Transcripts of the AMMI were scored by a trained member of our team. Six cases were double-coded by the AMMI’s creator, and interrater reliability was satisfying (83% of agreement).

The aforementioned measures have been validated in several languages including English. Their French version was used during this study.

#### Biometric Attachment Test

In order to evaluate our second hypothesis, we produced three BAT stimuli sets for this study: two fixed ones (ie, that show the same stimuli each time they are used) and a randomized one (ie, that shows different stimuli each time it is used). We have used the results from the French subsample of the survey respondents (n=194) to select the best pictures for a French population. The higher ranked pictures for each theme were put together in a stimuli set; the second higher ranked pictures were put together in a second stimuli set; and the pictures ranked third, fourth, and fifth were used to create a third set that randomly chooses one of those pictures each time it is played.

All 59 participants were exposed to the first stimuli set, 41 of them were also exposed to the second set, and 50 to the third rotating-stimuli set. Sets were presented one after the other.

#### Physiological Measures

HR was measured using the photoplethysmography sensor of an Empatica E4 wristband device. The sensor’s reliability has been established [63]. Like all heart sensors, the E4 is subject to artifacts produced by movement. Quality readings were obtained for 29 participants during the first BAT set, 19 during the second BAT set, and 9 during the third. Electrodermal activity (EDA), with a specific interest in skin conductance response (SCR), was measured using the EDA sensor of the Empatica E4 wristband device. Quality readings were obtained for all participants during all BATs. The EDA sensor’s reliability has been tested by the manufacturer [64]. Deliberately choosing to use a wireless wristband to measure physiological signals allowed our participants a more natural experience during the test.

#### Video and Audio Recording

Video of the participants’ faces was obtained through the computer’s webcam (Microsoft Surface Pro 4) and their speech was recorded using a USB Microphone (Samson GoMic). Since the BAT stimuli were presented using the same computer, gazing toward the stimuli was almost equivalent to gazing in the direction of the camera, facilitating gaze tracking.

### Feature Extraction

We conducted extensive feature extraction from each of the sense modalities captured during the BAT. All feature extraction procedures described below, including noise filtering processes, were performed programmatically without the need for human supervision.

The interbeat interval (IBI) was automatically calculated from the HR data by proprietary algorithms of the Empatica E4 research wristband [65]. The IBI files were cleaned of artifacts using Artiifact software, version 209 [66]. The same software was used for the extraction of heart rate variability (HRV) features (for a review of most standard HRV features, see [67]). We created a function in Microsoft Excel’s Visual Basic for Applications version 7.1 to automatically calculate Bayevsky stress index [54] from the IBI files.

From the EDA data, SCR, phasic maximal activity, and tonic skin conductance features were extracted using LedaLab software version 349 [68].

From the video data, facial expressions (such as anger and contempt, as well as the composites negative, neutral, and positive) were extracted using FACET’s Emotient [69]. A face size measure was extracted by the same software, which permits to establish the movement toward or away from the camera and thus the stimuli. Gaze direction was extracted using OpenFace [70].

From the audio recordings of the participants’ responses, paralinguistic acoustic features were extracted using the Cooperative Voice Analysis Repository for Speech Technologies (COVAREP) version 1.2 [71]. They help identify a breathy, relaxed voice from a tense voice.

We used Python and the French language model of Google’s Cloud Speech API to generate automated transcripts of all responses. We then processed the transcripts using Python and Linguistic Inquiry and Word Count (LIWC) French dictionary [72]. This dictionary is organized in 64 psychologically meaningful word categories. The frequency of each word category in the response to each theme was calculated, to be used as linguistic features.

Extracted features per theme were then treated in two different ways:

Subtracted baseline: results on the first theme (baseline theme) were subtracted from all other themes’ results. In theory, the resultant score should be more individualized to each person’s individual characteristics (eg, their specific mean HR baseline).

Subtracted previous theme: results on each theme were subtracted from the following one. In theory, the resultant score would isolate results from the exposure to the theme under analysis from the cumulative score due to exposure to all precedent themes (eg, the specific augmentation or decrease in mean HR when exposed to theme 7).

For many features, we further separated the reaction during exposure to the stimuli from the reaction during the verbal response to the stimuli, for example, facial expressions during exposure versus response.

Due to the high number of features extracted, the number of BAT themes, the two treatments we just described, and the separation between exposure and response, feature extraction led to a total of 4264 features per participant per stimuli set.

**Figure 2:**
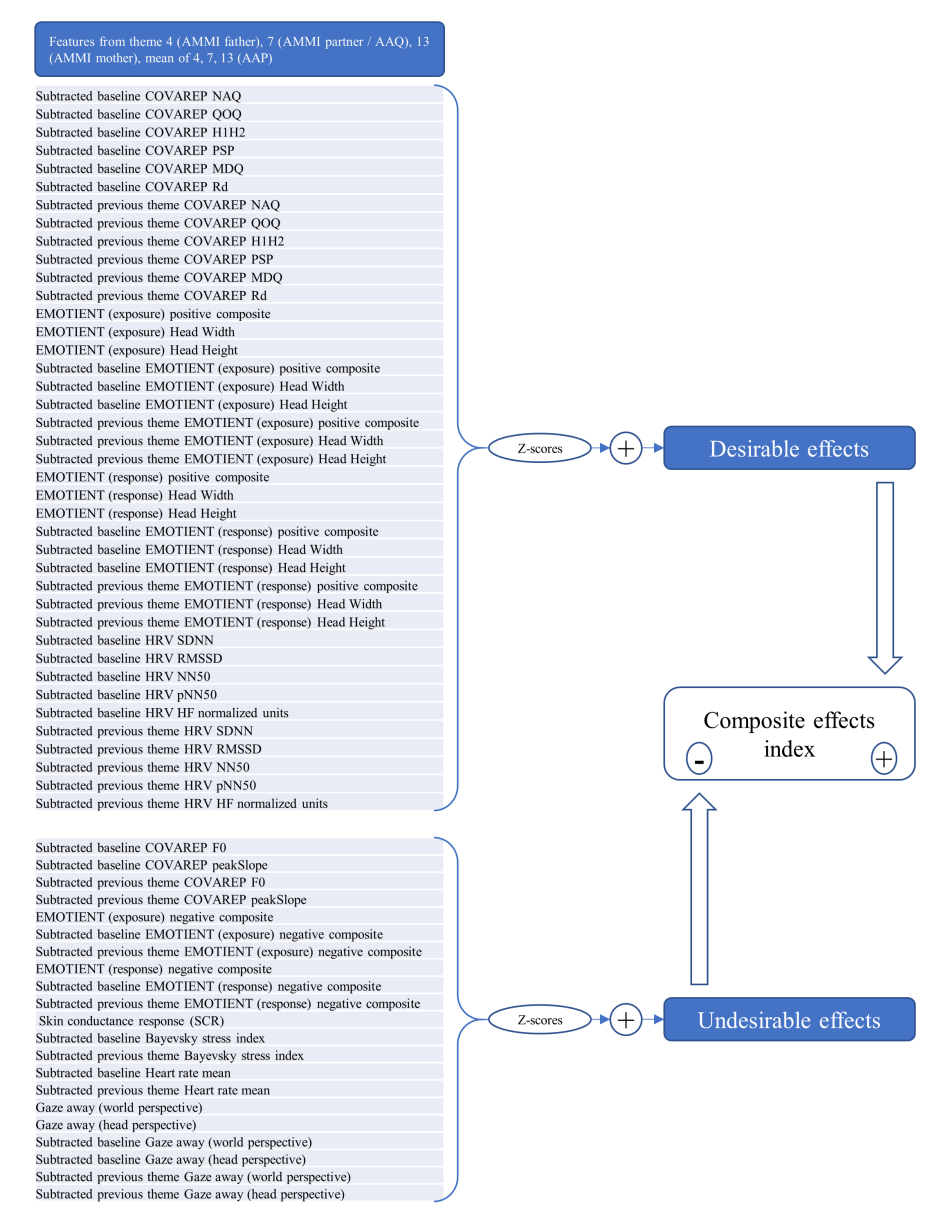
Development of the composite effects index.

In this study, we will focus on specific themes instead of the entire stimuli set, and each theme has 202 features (see Figure 2). In total, 2436 features pertain to the entire stimuli set as opposed to any single theme (eg, total stimuli-set-wise mean HR).

### Analyses

A first exploratory analysis, conducted in MathWorks Matlab version R2016A, consisted of performing correlation matrices to uncover associations between attachment security as measured by the AAQ, the AAP, and the AMMI, and the features extracted from BAT responses. For the AAQ and the AAP, we used Pearson correlations, whereas Spearman rank was used for the AMMI due to the small number of assessed participants. Since AAQ measures attachment to romantic partners, it was evaluated vis-à-vis theme 7 (“attuned couple”). AAP attachment security is concerned with attachment figures in general, thus we evaluated it with regards to a composite formed by the mean of responses to theme 4 (“attuned father-child”), theme 7 (“attuned couple”), and theme 13 (“attuned mother-child”). As per the AMMI, since it yields security scores for mother, father, and partner separately, we explored each in relationship to the corresponding BAT theme (themes 13, 4, and 7, respectively).

A second, confirmatory analysis was conducted using IBM SPSS Statistics version 23 to verify if our exploratory findings were not a mere spurious byproduct of multiple hypotheses testing [73]. We proceeded with a stringent approach consisting of producing a single “composite effects index” out of all available features (weighted in the same direction), then testing such index for a Pearson correlation vis-à-vis the variable of interest [74,75]. This approach circumvents the problem of type I errors often encountered in exploratory analyses. It also accounts for the problem of type II errors, which are likely when statistical correction procedures to control for family wise error rate (eg, Bonferroni correction) or false discovery rate (eg, Benjamini-Hochberg correction) are performed in studies with small samples, an elevated number of features, or small effect sizes such as ours [76-78].

For this analysis, our features’ scores were first transformed into *z* scores. Next, they were added to either an undesirable effects group or a desirable effects group. The decision was based on the literature available on each of the set of features, with the following results: the high frequency (HF) component of HRV (associated with parasympathetic “relaxing” activation), HRV’s SDNN, RMSSD, NN50, and pNN50 features (all of which convey slightly different aspects of the same, desirable construct: HRV), COVAREP features associated with a “breathy” relaxed voice, Emotient’s “positive emotions” composite, as well as the head size (proximity of participant to stimuli source), were all summed up within a desirable effects group. On the other hand, Bayevsky stress index, HR, gazing away from the stimuli, COVAREP features associated with a “tense” voice, Emotient’s “negative emotions” composite, as well as SCR levels were all summed up within an undesirable effects group. For each of the aforementioned features, scores extracted from the exposure phase and those from the response phase of the BAT were summed up (when available). Score treatments (subtracted baseline, subtracted previous theme) described earlier were also summed up, when available. Finally, a single composite effects index was created by subtracting the total score of the undesirable effects group from that of the desirable effects group. This index therefore is weighed in such a way that a higher score means more desirable effects and vice versa. Figure 2 illustrates this analysis.

Unfortunately, we could not include LIWC (linguistic features) in the analysis because they cannot easily be distributed among simple desirable or undesirable effects groups (eg, features such as “frequency of future tense verbs”). Finally, a few mathematically redundant (ie, equal information) features were omitted from this analysis, namely, the LF component of HRV in normalized units, as well as the HF/LF ratio of HRV (because their information is mathematically redundant with respect to the HF component in normalized units, see [79]); the percentage (%) and absolute power versions of the HF component feature (because the normalized units version of the feature controls for the very low frequency (VLF) component of HRV and thus is a more realistic measure of the same construct); the mean and median R-R features of HRV (because they are redundant with respect to HR). Specific EMOTIENT emotion features (eg, sadness) were not included separately since they are all included in two composites already produced by the software, one for negative expressions and the other for positive expressions. The Phasic maximal activity feature of EDA was not included for being redundant with respect to EDA’s SCR. The tonic skin conductance feature was not included because it requires longer measuring durations to be meaningful (they were calculated for future analyses focusing on the totality of the BAT instead of just isolated themes). The total amount of features per theme that ended being added up in the composite effects index is of 61 (see Figure 2).

A third analysis, conducted using IBM SPSS Statistics version 23, consisted in performing repeated measures analysis of variances (ANOVAs) on the BAT responses extracted features that were revealed as both statistically significant in their correlation to attachment security as well as theory consistent with attachment theory. The objective was to evaluate if those features yielded different results across different BAT stimuli sets or if they were consistently similar.

## Results

### Correlation Exploratory Analyses

#### Adult Attachment Questionnaire (AAQ; Pearson Correlations)

In the responses to BAT’s theme 7 (“attuned couple”), AAQ romantic attachment security was negatively correlated with negative facial expressions in general during exposure (*r*=−.32, *P*=.02) and anger in particular during response (*r*=−.43, *P*=.001) and exposure (*r*=−.38, *P*=.006). AAQ attachment security was also negatively correlated with the inhibition (r=−.38, *P*=.008), tentative (r=−.34, *P*=.02), and feeling (r=−.41, *P*=.004) categories of LIWC.

#### Adult Attachment Projective Picture System (AAP; Pearson Correlations)

In the responses to BAT’s theme 4 (“attuned father-child”), theme 7 (“attuned couple”), and theme 13 (“attuned mother-child”), using the mean of the responses to the three themes as a composite score, AAP attachment security was correlated with the NN50 after subtracting baseline (r=0.48, *P*=.007) and pNN50 after subtracting baseline (r=.38, *P*=.04), features of HRV, while it was negatively correlated with Bayevsky’s stress index after subtracting baseline (r=−.45, *P*=.01). AAP security was also correlated with the H1-H2 ratio of COVAREP after subtracting baseline (*r*=.30, *P*=.02). It was correlated as well with the hearing (*r*=.31, *P*=.02), we (*r*=.45, *P*<.001), leisure (*r*=.28, *P*=.04), and they (*r*=.40, *P*=.002) categories of LIWC. It was also correlated with positive facial expressions in general during exposure (*r*=.32, *P*=.01) and response (*r*=.36, *P*=.005) after subtracting previous theme, and joy in particular during response (*r*=.38, *P*=.003) after subtracting previous theme, as well as disgust in both exposure (r=.31, *P*=.02) and response (r=.33, *P*=.01) after subtracting previous theme. It was also negatively correlated with surprise on both exposure (r=−.29, *P*=.03) and response (r=−.32, *P*=.01), confusion during both exposure (*r*=−.27, *P*=.04) and response (*r*=−.27, *P*=.04), confusion during response (*r*=−.28, *P*=.03) after subtracting previous theme, anger during both exposure (*r*=−.37, *P*=.004) and response (*r*=−.31, *P*=.02) after subtracting previous theme, sadness during response (*r*=−.26, *P*=.04) after subtracting previous theme, neutral expressions during response (*r*=−.29, *P*=.02) after subtracting previous theme, fear during exposure after subtracting baseline (*r*=−.26, *P*=.05), and contempt during response (*r*=−.26, *P*=.05). It was negatively correlated with head size during exposure (*r*=−.32, *P*=.01) after subtracting previous theme and with head size during both exposure (*r*=−.37, *P*=.003) and response (*r*=−.35, *P*=.01) after subtracting baseline.

#### Attachment Multiple Model Interview (AMMI; Spearman Rank Correlations)

In the responses to BAT’s theme 4 (“attuned father-child”), AMMI father attachment security was correlated with the past tense (*r*=.47, *P*=.02), and negatively correlated with the discrepancy (*r*=−.46, *P*=.03) categories of LIWC. It was also negatively correlated with the LF feature of HRV after subtracting baseline (*r*=−.87, *P*=.03), and with SDNN feature of HRV after subtracting baseline (*r*=−.87, *P*=.03). It was also negatively correlated with head size in both exposure (*r*=−.43, *P*=.04) and response (*r*=−.45, *P*=.03). Finally, it was negatively correlated with facial expression of frustration during response (*r*=−.67, *P*<.001) and frustration after subtracting baseline during exposure (*r*=−.44, *P*=.04), as well as expressions of frustration (r=−.53, *P*=.01) and surprise (r=−.43, *P*=.04) after subtracting previous theme.

In the responses to BAT’s theme 13 (“attuned mother-child”), AMMI mother attachment security was correlated with SCR (*r*=.46, *P*=.02). It was correlated with facial expressions of sadness during exposure after subtracting baseline (*r*=.42, *P*=.03). It was negatively correlated with facial expression of sadness (r=−.51, *P*=.01) and with gazing away from the stimuli during exposure (r=−.41, *P*=.03) after subtracting previous theme.

In the responses to BAT’s theme 7 (“attuned couple”), AMMI partner attachment security was correlated with the high frequency feature of HRV in both normalized units (r=1, *P*=.02) and percentage (r=1, *P*=.02) after subtracting previous theme, and it was negatively correlated with the low frequency feature of HRV in both normalized units (r=−1, *P*=.02) and percentage (r=−1, *P*=.02) after subtracting previous theme, negatively correlated with the ratio of low versus high frequency of HRV (r=−1, *P*=.02) after subtracting previous theme, as well as negatively correlated with the mean heart rate (r=−1, *P*=.02). It was correlated with SCR (r=.61, *P*=.01). It was also correlated with face closeness to screen after subtracting baseline (*r*=.56, *P*=.02). It was negatively correlated with COVAREP Rd feature after subtracting baseline (*r*=−.60, *P*=.01). It was correlated with facial expressions of surprise after subtracting baseline (*r*=.59, *P*=.01). Finally, it was correlated with LIWC *exclusion* category (*r*=.49, *P*=.05).

### Confirmatory Analysis

Our composite effects index was significantly correlated to attachment security in the Adult Attachment Projective Picture System (*r*=.26, *P*=.05) by using the mean score from features of themes 4, 7, and 13 (like in the previous analysis), and significantly correlated to attachment security in the AAQ (*r*=.30, *P*=.04) by using scores from features of theme 7. Security with father, mother, and partner in the AMMI were unrelated to the composite effects index by using scores from features of themes 4, 13, and 7, respectively.

### Analysis of Variances (ANOVAs)

Repeated measures ANOVAs were conducted on each of the 46 features that were both statistically significant in their correlation to attachment security as well as theory consistent across the responses to the 3 BAT stimuli sets during the aforementioned specific themes. They revealed that only 7 (15%) of the 46 features had significantly different values depending on the stimuli set. Those features were the tentative category of LIWC during theme 7, *F*_1.56,49.92_=4.81, *P*=.02 (after a Greenhouse-Geisser correction); gazing away from the stimuli (after subtracting previous theme) during exposure to theme 13, *F*_2,76_=5.75, *P*=.005; and during themes 4, 7, and 13 (mean of the three), the hearing category of LIWC, *F*_2,64_=4.37, *P*=.02; the leisure category of LIWC, *F*_2,64_=4.63, *P*=.01; confusion facial expressions during exposure, *F*_1.7,66_=5, *P*=.01 (after a Greenhouse-Geisser correction) and during response, *F*_1.7,63_=6.3, *P*=.005 (after a Greenhouse-Geisser correction), as well as anger facial expressions during response after subtracting previous theme, *F*_1.7,62_=3.7, *P*=.04 (after a Greenhouse-Geisser correction).

## Discussion

### Principal Findings

Earlier in this work, we discussed the relevance that attachment theory has earned in mental health research, and we commented on the current limitations of psychometric instruments for assessing adult attachment.

We presented the BAT, a new adult-attachment assessment instrument, explicating its sources as well as its rationales and assumptions.

We then set to empirically evaluate two of the BAT’s core assumptions: that its themes can help measure attachment security as assessed by validated measures such as the AAQ, the AAP, and the AMMI; and that rotating the stimuli sets in the BAT would not alter the participants’ responses to the test.

Regarding the first hypothesis H1, during our exploratory analysis we were able to find physiological, behavioral, and linguistic markers of attachment security, both in general, and specific to romantic partners, mother, and father. These markers were elicited by the BAT’s specifically designed attachment-deactivating themes, which counts as preliminary evidence for the instrument’s internal and construct validity.

There was an important level of coherence, as well as theory consistency within our findings: in the presence of attachment-deactivating, reassuring stimuli, the more securely attached individuals experienced parasympathetic activation and sympathetic deactivation, a relaxation response revealed by increase in the HF and decrease in the LF of HRV [67], which also produced a decrease in overall stress as shown by the Bayevsky stress index [54]. During the verbal responses, the more securely attached participants’ voices became *breathier*, as revealed by COVAREP, indicating relaxation [71], and used more words that can convey attunement, like words related to hearing (eg, listen, heard) and the we pronoun, and conversely less words that can convey relational distress, like words related to inhibition (eg, block, constrain) and to tentativeness (eg, maybe, guess). Positive facial expressions, including joy, were related to attachment security, and their negative counterparts were mostly negatively correlated, as the theory would suggest. The more secure participants tended to not gaze away from the reassuring stimuli but, instead, got physically closer to them. Findings were not all theory consistent, however, as we’ll see below.

Since our exploratory analysis was based on multiple hypotheses testing, a statistical concern arose: could these findings be just the product of chance? But when all the available features, including the many that were not *significantly* correlated with attachment in the exploratory analyses, were summed up in a single composite effects index, said index was significantly correlated with two of our three attachment security “ground truth” measures, attesting to the robustness of the findings. This analysis might also suggest that some of the features not showing a statistically significant association with attachment security might not achieve so because of a small sample size or small effect sizes. Composites help increase the effect size of features weighting in the same direction, statistically revealing their direction [74].

We believe this sort of multimodal automatic appraisal of “the whole picture” that is an attachment-deactivating reaction, from a behavioral, psychophysiological, and linguistic standpoint, is a taste of what is becoming possible for psychometrics. Moreover, the fact that our data was obtained outside of a lab setting, using a consumer tablet and its webcam, a consumer-grade USB microphone, and a wireless wristband, attests to the pace at which sensing technology is advancing, offering a glimpse at how effortlessly these measures could be obtained in a close future.

Regarding the second hypothesis H2, 84% (39/46) of the features revealed as associated with attachment security and that were theory consistent were stable across three different BAT stimuli sets. This is especially remarkable given that one of those stimuli sets (always presented second in order) was randomized every time the test was administrated, which means that no person, among our sample, saw or listened to the exact same second stimuli set. This finding suggests that during the BAT, participants react and respond mainly to the themes (ie, the attachment narratives) which are being evoked by the stimuli, and not so much to the stimulus details themselves (eg, the color of a person’s hair or the specific background). This also suggests that, as long as stimuli are selected using the standardized procedure described in this work and our minimum fitness scores are respected, new stimuli sets could be developed for the BAT without affecting its capacity to evoke and measure attachment. H2 results suggest, of course, that we should stop including the 7 features that did change across stimuli sets in further developments of the BAT’s scoring algorithms, as they seem to be less reliable when stimuli sets are varied.

We have chosen not to perform multiple comparison corrections (eg, Bonferroni) in our repeated measures ANOVAs to, counterintuitively, increase the rigor of the analysis. This is because within our ANOVA analysis, we compared 46 features across 3 different stimuli sets, for a total of 138 *F* tests. A Bonferroni correction would imply that the alpha level is divided by the number of comparisons (.05/138) for a corrected alpha level of .0003. It would be very difficult for any difference to be found under this alpha level with our sample size. This would be a convenient result, but probably a false one.

### Limitations

Out of the 65 significant correlations revealed by our exploratory analysis, 19 (29%) seemed to go against what would be expected from an attachment theory perspective. Some of the most striking examples were the increase in sadness expressions correlated with AMMI mother model security, or the increase of disgust expressions correlated with AAP security. Thus when developing our scoring and classification algorithms in the future, it will be important to discard such features, unless we can find theoretical underpinnings for them.

The confirmatory analysis we performed, based on creating a composite effects index, was designed to prove that *overall* the many features studied when put together weighted in the right direction in correlation with attachment security. We argue that this is confirmatory evidence for the construct validity of the BAT, namely that the test activates people in a way that can be captured by multiple modalities and that is correlated with attachment security; but we do not present this as confirmatory evidence for the relationship of any specific feature and attachment security. For example, our study suggests, but does not confirm, that a breathier voice can be a watermark of a more securely attached person during an attachment-soothing situation. In this sense, specially associations of LIWC features and attachment security should be seen as merely exploratory since LIWC was not included in our confirmatory analysis (for a rigorous confirmatory study about LIWC features and attachment, see [80]). As for security in the AMMI, which was not significantly correlated with our composite effects index (albeit it was with several individual features), it is important to restate that only a fraction of our sample took that test (n=27 for the mother model, n=23 for the father model, n=17 for the partner model) and since the effect sizes of most features are small, this might explain the lack of association.

In our ANOVA analysis, we decided to include only features that were found to be both statistically significant in their associations to attachment security as well as theory consistent in that association. Why? We compared incredibly different variables in these ANOVAs; from word count on a variety of categories to facial expressions to HRV, and so on, as a reaction to very different image and music combinations. All these features were calculated without human supervision out of the raw input data, introducing some degree of random error that should favor a finding of difference between answers to different stimuli sets. As a result, odds were stacked in favor of finding differences, and avoiding a multiple comparison correction made it more so as explained above. Including more features (eg, theory-inconsistent features) without controlling for multiple comparisons would generate just too many type I errors for the analysis to be useful.

### Future Directions

The BAT was designed to test far more than the attachment security dimension. The different themes in the BAT were designed to also test for the other three main attachment dimensions: attachment anxiety, attachment avoidance [40], and attachment disorganization [12]. They were also designed to measure attachment defenses, such as deactivation, cognitive disconnection, and segregated systems [13]. Different themes heighten differences in the reactions of the four classic adult attachment groups (dismissing, preoccupied, secure, unresolved) to help in classification of attachment. Finally, some themes in the BAT were designed to measure emotional regulation, as measured by instruments such as the Difficulties in Emotion Regulation Scale (DERS [81]), and relational trauma, as measured by the trauma system of the AAP [13]. Empirical validation of the BAT’s fitness to measure these constructs is, of course, warranted.

An important area of our work with the BAT is the development of algorithms to automatically score and classify attachment based on extracted features from responses to the test, like the ones highlighted by this study.

This endeavor is complex. It entails finding the right fusion formula for the different BAT features so that the emerging multimodal pattern can accurately predict attachment continuous scores and classifications. It also entails extensive cross-validation to verify the generalizability of the prediction capability to new cohorts. We are underway in this work, and in fact we have developed preliminary regression and classification algorithms capable of predicting ground truth attachment continuous scores and classifications better than chance, cross-validating our results to prevent over-fitting and to warrant generalizability.

### Conclusions

Overall, this study brings us one step closer to our goal of developing an automatic and objective adult attachment test. In the future, a 9-min BAT test could be deployed through the Internet to participants or patients residing in remote areas. The test could be scored instantaneously and automatically, with the results becoming available to the researcher or clinician just minutes later. We hope that this could unleash a new wave of attachment research as well as favor clinical attachment testing, in turn benefiting patients by offering them more cost-effective and efficient mental health assessments and treatments.

## Abbreviations

AAI: Adult Attachment Interview
AAP: Adult Attachment Projective Picture System
AAQ: Adult Attachment Questionnaire
AAS: Adult Attachment Scale
AMMI: Attachment Multiple Model Interview
ANOVA: analysis of variance
ARL: Army Research Laboratory
BAT: Biometric Attachment Test
COVAREP: Cooperative Voice Analysis Repository for Speech Technologies
DERS: Difficulties in Emotion Regulation Sale
EDA: electrodermal activity
GAPED: Geneva Affective Picture Database
HF: high frequency
HR: heart rate
HRV: heart rate variability
IAPS: International Affective Picture System
IBI: interbeat interval
LF: low frequency
LIWC: Linguistic Inquiry and Word Count
NAPS: Nencki Affective Picture System
PTSD: posttraumatic stress disorder
SAT: Separation Anxiety Test
SCR: skin conductance response
SPT: Standard Penetration Test
SSP: Strange Situation Procedure
VLF: very low frequency

## References

[1] Bowlby J (1969). Attachment and loss.

[2] Cassidy J, Shaver P (2016). editors. Handbook of attachment: Theory, research, and clinical applications. 3rd edition.

[3] Sroufe LA, Waters E (1977). Attachment as an Organizational Construct. Child Development.

[4] Mesman J, van IJzendoorn MH, Sagi-Schwartz A, Cassidy J, Shaver PR (2016). Cross-cultural patterns of attachment: universal and contextual dimensions. Handbook of Attachment: Theory, Research, and Clinical Applications.

[5] Grossmann K, Grossmann K, Keppler A, Friedlmeier W, Chakkarath P, Schwarz B (2005). Universal and Culture-Specific Aspects of Human Behavior: The Case of Attachment. Culture and Human Development: The Importance of Cross-Cultural Research to the Social Sciences.

[6] Ainsworth MD (1985). Attachments across the life span. Bull N Y Acad Med.

[7] Sroufe LA, Coffino B, Carlson EA (2010). Conceptualizing the role of early experience: lessons from the minnesota longitudinal study. Dev Rev.

[8] Fraley C, Roisman G (2015). Do early caregiving experiences leave an enduring or transient mark on developmental adaptation?. Curr Opin Psychol.

[9] De Wolff MS, van IJzendoorn MH (1997). Sensitivity and attachment: a meta-analysis on parental antecedents of infant attachment. Child Development.

[10] Lorber MF, Egeland B (2009). Infancy parenting and externalizing psychopathology from childhood through adulthood: developmental trends. Dev Psychol.

[11] Miljkovitch R, Moran G, Roy C, Jaunin L, Forcada-Guex M, Pierrehumbert B, Muller-Nix C, Borghini A (2013). Maternal interactive behaviour as a predictor of preschoolers' attachment representations among full term and premature samples. Early Hum Dev.

[12] Miljkovitch R, Moss E, Bernier A, Pascuzzo K, Sander E (2015). Refining the assessment of internal working models: the Attachment Multiple Model Interview. Attach Hum Dev.

[13] George C, West M (2012). The adult attachment projective picture system: attachment theory and assessment in adults.

[14] Mikulincer M, Dolev T, Shaver PR (2004). Attachment-related strategies during thought suppression: ironic rebounds and vulnerable self-representations. J Pers Soc Psychol.

[15] Coan J, Cassidy J, Shaver PR (2016). Towards a neuroscience of attachment. Handbook of Attachment: Theory, Research, and Clinical Applications.

[16] Hane A, Fox N, Cassidy J, Shaver PR (2016). Studying the biology of human attachment. Handbook of Attachment: Theory, Research, and Cinical Applications.

[17] Lemche E, Giampietro VP, Surguladze SA, Amaro EJ, Andrew CM, Williams SC, Brammer MJ, Lawrence N, Maier MA, Russell TA, Simmons A, Ecker C, Joraschky P, Phillips ML (2006). Human attachment security is mediated by the amygdala: evidence from combined fMRI and psychophysiological measures. Hum Brain Mapp.

[18] Fraley RC, Spieker SJ (2003). Are infant attachment patterns continuously or categorically distributed? a taxometric analysis of strange situation behavior. ‎Dev Psychol.

[19] Grossmann KE, Grossmann K, Waters E (2005). Attachment from Infancy to Adulthood: The Major Longitudinal Studies.

[20] Carlson EA (1998). A prospective longitudinal study of attachment disorganization/disorientation. Child Development.

[21] Mikulincer M, Shaver PR (2012). An attachment perspective on psychopathology. World Psychiatry.

[22] Bakermans-Kranenburg MJ, van IJzendoorn MH (2009). The first 10,000 adult attachment interviews: distributions of adult attachment representations in clinical and non-clinical groups. Attach Hum Dev.

[23] Cantazaro A, Wei M (2010). Adult attachment, dependence, self-criticism, and depressive symptoms: a test of a mediational model. J Pers.

[24] Ein-Dor T, Doron G, Solomon Z, Mikulincer M, Shaver PR (2010). Together in pain: attachment-related dyadic processes and posttraumatic stress disorder. J Couns Psychol.

[25] Levy KN, Meehan KB, Weber M, Reynoso J, Clarkin JF (2005). Attachment and borderline personality disorder: implications for psychotherapy. Psychopathology.

[26] Brown DP, Elliott DS (2016). Attachment disturbances in adults: Treatment for comprehensive repair.

[27] Mikulincer M, Shaver PR (2015). The psychological effects of the contextual activation of security-enhancing mental representations in adulthood. Curr Opin Psychol.

[28] Parnell L, Siegel DJ (2015). Attachment-focused EMDR: Healing relational trauma.

[29] Mikulincer M, Shaver P, Cassidy J, Shaver PR (2016). Adult attachment and emotion regulation. Handbook of Attachment: Theory, Research, and Clinical Applications.

[30] Bergman K, Sarkar P, Glover V, O'Connor TG (2010). Maternal prenatal cortisol and infant cognitive development: moderation by infant-mother attachment. Biol Psychiatry.

[31] Miljkovitch R (2009). Les fondations du lien amoureux.

[32] Feeney J, Cassidy J, Shaver PR (2016). Adult romantic attachment: developments in the study of couple relationships. Handbook of Attachment: Theory, Research, and Clinical Applications.

[33] Rodin G, Walsh A, Zimmermann C, Gagliese L, Jones J, Shepherd FA, Moore M, Braun M, Donner A, Mikulincer M (2007). The contribution of attachment security and social support to depressive symptoms in patients with metastatic cancer. Psychooncology.

[34] Ditzen B, Schmidt S, Strauss B, Nater UM, Ehlert U, Heinrichs M (2008). Adult attachment and social support interact to reduce psychological but not cortisol responses to stress. J Psychosom Res.

[35] Bartholomew K, Horowitz LM (1991). Attachment styles among young adults: a test of a four-category model. J Pers Soc Psychol.

[36] Larose S, Bernier A (2001). Social support processes: mediators of attachment state of mind and adjustment in late adolescence. Attach Hum Dev.

[37] Powers SI, Pietromonaco PR, Gunlicks M, Sayer A (2006). Dating couples' attachment styles and patterns of cortisol reactivity and recovery in response to a relationship conflict. J Pers Soc Psychol.

[38] Wallace JL, Vaux A (1993). Social support network orientation: the role of adult attachment style. J Soc Clin Psychol.

[39] Ravitz P, Maunder R, Hunter J, Sthankiya B, Lancee W (2010). Adult attachment measures: a 25-year review. J Psychosom Res.

[40] Simpson JA, Rholes WS, Phillips D (1996). Conflict in close relationships: An attachment perspective. J Pers Soc Psychol.

[41] Collins NL, Read SJ (1990). Adult attachment, working models, and relationship quality in dating couples. J Pers Soc Psychol.

[42] Podsakoff PM, MacKenzie SB, Lee J, Podsakoff NP (2003). Common method biases in behavioral research: a critical review of the literature and recommended remedies. J Appl Psychol.

[43] Ainsworth MDS, Bell SM (1970). Attachment, exploration, and separation: illustrated by the behavior of one-year-olds in a strange situation. Child Development.

[44] Cassidy J, Sherman LJ, Jones JD (2012). What's in a word? linguistic characteristics of adult attachment interviews. Attach Hum Dev.

[45] Maxwell SE, Lau MY, Howard GS (2015). Is psychology suffering from a replication crisis? what does “failure to replicate” really mean?. Am Psychol.

[46] Cummins N, Scherer S, Krajewski J, Schnieder S, Epps J, Quatieri TF (2015). A review of depression and suicide risk assessment using speech analysis. Speech Commun.

[47] Scherer S, Gale L, Gratch J, Rizzo A, Morency L-P (2016). Self-reported symptoms of depression and PTSD are associated with reduced vowel space in screening interviews. IEEE Trans Affective Comput.

[48] (2013). NIMH.

[49] Scherer S, Stratou G, Lucas G, Mahmoud M, Boberg J, Gratch J, Rizzo A(, Morency L (2014). Automatic audiovisual behavior descriptors for psychological disorder analysis. Image Vis Comput.

[50] Novak D, Mihelj M, Munih M (2012). A survey of methods for data fusion and system adaptation using autonomic nervous system responses in physiological computing. Interact Comput.

[51] Lucas G, Gratch J, Scherer S, Boberg J, Stratou G (2015). Towards an affective interface for assessment of psychological distress.

[52] Klagsbrun M, Bowlby J (1976). Responses to separation from parents: a clinical test for young children. British Journal of Projective Psychology & Personality Study.

[53] Baumgartner T, Lutz K, Schmidt CF, Jäncke L (2006). The emotional power of music: how music enhances the feeling of affective pictures. Brain Res.

[54] Bayevsky RM (2002). Analysis of heart rate variability in space medicine. Hum Physiol.

[55] Tuber S (2014). Understanding personality through projective testing.

[56] Schultz DS, Loving JL (2012). Challenges since Wikipedia: the availability of Rorschach information online and internet users' reactions to online media coverage of the Rorschach-Wikipedia debate. J Pers Assess.

[57] Marchewka A, Zurawski L, Jednoróg K, Grabowska A (2014). The Nencki Affective Picture System (NAPS): introduction to a novel, standardized, wide-range, high-quality, realistic picture database. Behav Res Methods.

[58] Lang P, Bradley M, Cuthbert B (1999). International affective picture system (IAPS): Technical manual and affective ratings.

[59] Dan-Glauser ES, Scherer KR (2011). The Geneva affective picture database (GAPED): a new 730-picture database focusing on valence and normative significance. Behav Res Methods.

[60] Zapin M SurveyGizmo.

[61] Eerola T, Vuoskoski JK (2010). A comparison of the discrete and dimensional models of emotion in music. Psychol Music.

[62] Mathôt S, Schreij D, Theeuwes J (2012). OpenSesame: an open-source, graphical experiment builder for the social sciences. Behav Res Methods.

[63] McCarthy C, Pradhan N, Redpath C, Adler A (2016). Validation of the Empatica E4 wristband.

[64] (2015). Empatica.

[65] Garbarino M, Lai M, Bender D (2014). Empatica E3 A wearable wireless multi-sensor device for real-time computerized biofeedback and data acquisition.

[66] Kaufmann T, Sütterlin S, Schulz SM, Vögele C (2011). ARTiiFACT: a tool for heart rate artifact processing and heart rate variability analysis. Behav Res Methods.

[67] Task Force of the European Society of Cardiology, The North American Society of Pacing and Electrophysiology (1996). Heart rate variability: standards of measurement, physiological interpretation and clinical use. Circulation.

[68] Benedek M, Kaernbach C (2010). A continuous measure of phasic electrodermal activity. J Neurosci Methods.

[69] Littlewort G, Whitehill J, Wu T, Fasel I, Frank M, Movellan J, Bartlett M (2011). The computer expression recognition toolbox (CERT). Gesture Recognition.

[70] Amos B, Ludwiczuk B, Satyanarayanan M (2016). ADM.

[71] Degottex G, Kane J, Drugman T, Raitio T, Scherer S (2014). COVAREP: A collaborative voice analysis repository for speech technologies.

[72] Piolat A, Booth R, Chung C, Davids M, Pennebaker J (2011). La version française du dictionnaire pour le LIWC : modalités de construction et exemples d’utilisation. Psychologie Française.

[73] Bender R, Lange S (2001). Adjusting for multiple testing-when and how?. J Clin Epidemiol.

[74] Anderson ML (2008). Multiple inference and gender differences in the effects of early intervention: a reevaluation of the abecedarian, perry preschool, and early training projects. J Am Stat Assoc.

[75] Kling JR, Liebman JB, Katz LF, Sanbonmatsu L (2004). Princeton.

[76] Rothman KJ (2014). Six persistent research misconceptions. J Gen Intern Med.

[77] Pascovici D, Handler DC, Wu JX, Haynes PA (2016). Multiple testing corrections in quantitative proteomics: a useful but blunt tool. Proteomics.

[78] Feise RJ (2002). Do multiple outcome measures require p-value adjustment?. BMC Med Res Methodol.

[79] Burr RL (2007). Interpretation of normalized spectral heart rate variability indices in sleep research: a critical review. Sleep.

[80] Waters TE, Steele RD, Roisman GI, Haydon KC, Booth-LaForce C (2016). A linguistic inquiry and word count analysis of the adult attachment interview in two large corpora. Can J Behav Sci.

[81] Gratz KL, Roemer L (2004). Multidimensional assessment of emotion regulation and dysregulation: development, factor structure, and initial validation of the difficulties in emotion regulation scale. J Psychopathol Behav Assess.

